# Polar Microbiology: Recent Advances and Future Perspectives

**DOI:** 10.3390/biology3010081

**Published:** 2014-02-03

**Authors:** Pabulo Henrique Rampelotto

**Affiliations:** Interdisciplinary Center for Biotechnology Research, Federal University of Pampa, AntônioTrilha Avenue, P.O.Box 1847, 97300-000, São Gabriel – RS, Brazil; E-Mail: pabulo@lacesm.ufsm.br

Most of the Earth’s biosphere is cold (85% is permanently exposed to temperatures below 5 °C), with 14% being polar. Polar regions are areas of the globe surrounding the poles. They are different in that the Arctic is a frozen ocean surrounded by continental landmasses and open oceans, whereas Antarctica is a frozen continent surrounded solely by oceans. The Arctic has numerous definitions; the most used defines it as the region north of the Arctic Circle (66.5° north latitude), including the Arctic Ocean and the islands and northern continental land areas from North America, Europe and Asia. According with the 1959 Antarctic Treaty, Antarctica is defined as the region south of 60° south latitude. Both polar regions are dominated by cold conditions and the presence of ice, snow, and water. Nonetheless, these environments are far from uniform and present a great variety of niches including different rocks, sediments, soil types, and melt-waters, as well as snow and ice that vary in terms of nutrient and water availability, salinity, and thermal regime. Even the apparently uniform environment of ice contains a network of liquid water veins (brine veins) that can transport soluble and insoluble particles and support life within it. Novel studies have demonstrated that active microbial respiration occurs within these ice structures and that there may have significant microbial variability within these ice-rich environments.

This diversity of polar environments is reflected in a wide diversity of cold adapted microorganisms which include members of the three domains of life, i.e., Bacteria, Archaea and Eukarya. Together they cover a wide range of nutritional types including aerobes and anaerobes, heterotrophs and autotrophs as well as chemolithotrophs and chemoautotrophs. In addition, polar regions also possess unusual microbiotopes such as the porous rocks in Antarctic Dry Valleys hosting microbial communities that survive at −60 °C, the liquid brine veins between sea ice crystals harbor metabolically active microorganisms at −20 °C, and permafrost cryopegs, i.e., salty water pockets that have remained liquid at −10 °C for about 100,000 years.

One of the most groundbreaking discoveries about cold environments in recent years was the unexpected finding beneath the Antarctic ice cap, a system of rivers and lakes which has been separated from the surrounding world for hundreds of thousands of years or longer. This exciting and unexpected discovery of sub-glacial lakes and rivers has profound implications for our understanding of biology. These environments represent unique opportunities for exploring new forms of life adapted to extreme conditions and evolving in the absence of gene flow from outside biota, resulting in unique metabolically active microbial assemblages in terms of structure and function, with the potential to provide new insights into microbial evolution.

Although several investigations studied in depth the eukaryote organisms during the last years, little is known about the biology of microorganisms in cold environments, especially in polar regions. However, this scenario is changing. The advent of the genomic era allowed us to investigate intriguing questions on the nature of cold adapted microorganisms with unprecedented precision. In particular, the new high-throughput DNA sequencing technologies have revolutionized the exploration of polar microbiology revealing microbial ecosystems with unexpectedly high levels of diversity and complexity. Nevertheless, the understanding of the functional roles of microorganisms in the carbon cycling, nitrogen and other elements/materials in cold ecosystems continues to rely upon more traditional methods, albeit applied with new insight. Consequently, the combination of both culture-dependent and culture-independent techniques has been considered the best approach towards a better understanding of how microorganisms survive and function in such extreme environments. These multidisciplinary approaches and technologies provide new pathways into a new frontier of research opportunities at the Poles.

Nowadays, polar microbiology is a promising field of research that can tell us much about the fundaments of life. The microorganisms that inhabit polar regions are important not only because of the unique species they represent, but also because of their diverse and unusual physiological/biochemical properties. The mechanisms by which different microorganisms adapt to the extreme cold environment offer powerful study systems for elucidating the fundamental properties of cellular design and the ways in which evolutionary changes in the cell adapts organisms to their environments. Previous studies have revealed various adaptations to cold conditions at the molecular and cellular level such as the synthesis of antifreeze proteins and cold-active enzymes or the incorporation of membrane unsaturated fatty acids promoting homeoviscosity. A better understanding of these complex adaptations will be achieved by multidisciplinary analysis encompassing genomics, proteomics, and transcriptomics. Furthermore, microorganisms living in polar regions provide useful models for general questions in ecology and evolutionary biology. Given their relative isolation (especially in Antarctica), the reduced complexity of their ecosystems, the relative absence of confounding effects associated with higher plants or animals, and the severe biological constraints imposed by the polar environment. 

Another compelling reason to study polar microbial ecosystems is that they are likely to be among the ecosystems most strongly affected by global changes. Polar regions are experiencing the earliest and most pronounced changes from global warming. Recent estimates indicate that the Arctic is warming twice as fast as other parts of the world. In the opposite hemisphere, the Antarctic Peninsula has also warmed rapidly; this region has experienced increases of 3 °C in the annual mean temperature and 6 °C in the winter temperature over the last 50 years. The polar amplification of global warming leads to systemic alterations in the regional environment like rapid decline of the sea-ice cover, thawing of the permafrost as well as melting of ice sheets and glaciers. These changes may have dramatic impact on the polar microbial ecosystems (which are highly sensitive to changes due the severe biological constraints imposed by the polar environment) including significant shifts in their abundance, structure and function as well as potential extinction of several species. In addition, warmer conditions may also be more favorable to invasive species brought by natural processes and by the increasing human activity in these regions. Consequently, these unique microbial ecosystems may now be facing imminent extinction. Indeed, some polar microbial ecosystems appear to be in rapid decline, whereas others are shifting towards new states, with implications for food webs and biogeochemical fluxes. However, the responses of microbial ecosystems to climate change are complex and subject to interactions and feedbacks. For these reasons, further studies are necessary to characterize in more details the impacts of these environmental changes on polar microorganisms at all levels of biological organization (i.e., from biochemistry and molecular biology to physiology and ecology).

Furthermore, climatic changes in polar regions are likely to generate globally significant cascades that will affect all life on Earth. A worrying example comes from the thawing of permafrost and the release of greenhouse gases. Permafrost stores an immense amount of carbon (twice as much carbon as contained in the atmosphere) that has accumulated over thousands of years. As a result of climate change, the thawing of permafrost would release the stored organic carbon in the form of carbon dioxide and methane, increasing the concentration of these greenhouse gases in the atmosphere, which would dangerously accelerate global warming.

Microbiological studies in polar regions are also particularly relevant to astrobiology and the search for life in our Solar System due the existence of habitat analogues for the Martian polar ice caps (Arctic tundra and Antarctic desert soils) and the Jupiter's icy moon Europa (Lake Vostok and other perennially subglacial lakes from Antarctica). In this way, our increasing knowledge on the molecular and cellular adaptation of microorganisms to cold environments on Earth has the potential to provide useful information on how life might develop and survive under extreme conditions on other planets or moons. These cold terrestrial environments have also been used to test life-detection methods and instruments to be used in future missions on other planetary icy bodies. 

In terms of applied science, the uniquely cold-adapted enzymes and other biomolecules of polar microorganisms provide numerous opportunities for biotechnological development. The biotechnology based on polar genetic and molecular aspects covers several key areas including enzymes, anti-freeze proteins, bioremediation, pharmaceuticals and other health related applications. Indeed, bioprospecting at the Poles has increased in recent years drawing significant attention from several industrial sectors and biotech companies for commercially-exploitable activities.

For these reasons, polar microbiology is a thriving branch of science with the potential to provide new insights into a wide range of basic and applied issues in biology. In this context, it is timely to review and highlight the progress so far and discuss exciting future perspectives. In this special issue, some of the leaders in the field have described their work, ideas and findings in a collection of reviews and original research articles with studies ranging from one of the oldest permafrost areas on Earth, located in Siberia, to the accretion ice of Lake Vostok, located in Antarctica. Altogether, these articles provide a comprehensive and reliable source of information on the current advances and future perspectives in this exciting field of research.

I would like to thank the authors and co-authors for submitting such interesting contributions as well as the Editorial Office and numerous reviewers for their valuable assistance in reviewing the manuscripts. 

